# Outcome of TACE treatment in HIV infected patients with hepatocellular carcinoma

**DOI:** 10.1038/s41598-020-80311-3

**Published:** 2021-01-12

**Authors:** Lingxiang Kong, Guo Wei, Tao Lv, Li Jiang, Jian Yang, Yong Zhao, Jiayin Yang

**Affiliations:** 1 Department of Liver Surgery, West China Hospital of Sichuan University, Chengdu, Sichuan Province China; 2 Department of General Surgery, Chengdu Public Health Clinical Medical Center, Chengdu, Sichuan Province China

**Keywords:** Hepatology, Surgical oncology, Hepatitis, HIV infections, Gastrointestinal cancer

## Abstract

The surgical treatment and transcatheter arterial chemoembolization (TACE) rate of human immunodeficiency virus (HIV)-infected hepatocellular carcinoma (HCC) patients is relatively low in West China. For various reasons, most patients do not receive timely surgical treatment. Upon transfer to an infectious disease centralized hospital, they were already classified in the Barcelona Clinic Liver Cancer (BCLC)-B stage. A total of 2249 BCLC-B HCC patients were analyzed. The eligible population was divided into three groups for analysis of survival and prognostic factors; These were 21 HIV infected (HIV+) HCC patients treated with TACE (TACE+), 1293 non-HIV-infected (HIV−) HCC patients treated with TACE, and 150 HIV− HCC patients who only receive medication (TACE−) as a second control group. After 1:2 matching, 1- and 2-year survival of HIV+ TACE+ and HIV− TACE+ groups was 64.3% and 76.5% (*P* = 0.453) and 45.5% vs. 50.0% (*P* = 0.790) respectively. We also compared one and two-year survival between HIV+ TACE+ and HIV− TACE−. One-year overall survival was 64.3% vs. 45.7% (*P* = 0.097) and 2-year survival was 45.5% vs. 7.1% (*P* = 0.004). Multivariate analysis showed that the most important prognostic factors for survival were serum alpha-fetoprotein (AFP) and Child–Pugh score and tumor size, while HIV status had no significant effect on prognosis statistically. CD4 levels below 200 may increase the risk of opportunistic infection after surgery, but after anti-infection and systematic supportive therapy, it has no effect on survival. HIV+ patients should have the same treatment opportunities as HIV− patients. If the patient's immune status permits, we suggest that early TACE treatment should be administered to BCLC-B HCC patients, regardless of HIV infection.

## Introduction

As of September 2016, the estimated number of HIV+ patients in China is approximately 960,000, and is increasing year by year. Since the introduction of highly active antiretroviral treatment (HAART), patients with HIV have life expectancy comparable to that of the general population^1,2^. Liver disease has now become one of the leading causes of hospitalization and death in HIV+ patients, HCC being the main reason^3^.

In China, the treatment of HIV+ patients is concentrated in infectious disease hospitals, which have limited capacity to carry out major operations such as liver resection or transplantation. Staff in general hospitals are generally concerned about HIV exposure, and the implementation of surgical treatment for HIV is therefore challenging. As a result, HIV+ patients with HCC are often only treated with medication. This also leads to a low rate of radical surgery in HIV+ patients even if HCC is detected early, and these patients will eventually enter the BCLC-B stage due to the natural progression of HCC. In addition, because of ignorance concerning HIV, many patients still believe it to be a terminal disease; consequently, patients often lose hope and neglect further medical care. Even if HCC is diagnosed, persuading patients to undergo surgery and TACE is difficult. The vast majority of HIV+ patients in our hospital are also BCLC-B patients, most of whom have had no surgery prior to transfer.

TACE is a non-curative treatment, to some extent, minimally invasive surgery, and it is also the best recommendation for BCLC-B HCC patients^4^. Numerous studies have confirmed that patients with unresectable HCC have survival benefits from TACE^5,6^. Not only does TACE have a bridging therapeutic function, i.e., controlling HCC development while awaiting liver transplantation, but it can also be used as step-down therapy for HCC. TACE as a minimally invasive treatment compared with traditional surgery eliminates the risk of HIV exposure and has a high safety factor. Therefore, the main purpose of this study was to discuss survival and prognostic factors of TACE in the treatment of HCC patients with HIV.

## Methods

Figure S1 details the study design and patient grouping. HCC was diagnosed and managed according to the European Association for the Study of the Liver guidelines^7^, American Association for the Study of Liver Diseases updated practice guidelines^8^, and the Barcelona Clinic of Liver Cancer guidelines^9^.

A literature search was conducted via PubMed using the following terms and strategy to find the relevant studies: (“HIV”) AND (“HCC” or “hepatocellular carcinoma”). The inclusion criteria for the eligible studies were: (1) Prospective and retrospective studies that assessed the safety and efficacy of HCC patients with or without HIV infection; (2) Studies reporting on overall survival status for at least two years after treatment.

SPSS 23.0 was used to analyze the relevant data. Independent factors for the platelet count and overall survival were analyzed using Cox proportional hazards models. *P* < 0.05 was considered to be statistically significant. RR was 0.8–1 or 1.0–1.2, indicating that there was no correlation between exposure factors and disease. Propensity score matching was used to provide a one-to-two nearest-neighbor match between the experimental and control group. Matching conditions were: age; sex; body mass index (BMI); hepatitis B status; Child–Pugh Score; total bilirubin (TB); hemoglobin (HB); white blood cell count (WBC); creatinine (Cre); aspartate aminotransferase (AST); alanine aminotransferase (ALT); platelet count (Plt); albumin (Alb); Prothrombin time (PT), AFP > 400 ng/mL, number of tumors and maximum diameter of tumors. The Review Manager 5.3 was used for data pooling. The end points of this meta-analysis were 1- and 2-year overall survival status. The results of the data pooling in the meta-analysis were presented as forest plots.

### Ethical approval

The protocol was approved by the Ethics Committee of the West China Hospital of Sichuan University West China Hospital. Written informed consent was obtained from all recipients prior to surgery, and all patients entered the study voluntarily, in accordance with the Declaration of Helsinki.

## Results

### Univariate and multivariate analysis via Cox regression

Table 1 shows the univariate analysis of various factors for BCLC-B HCC patients in the overall sample and HIV+ patients separately. A total of 1464 BCLC-B patients who met the inclusion criteria from January 2001 to December 2018 were included in this single factor analysis. TB, ALT and PT had statistical significance in univariate analysis (*P* < 0.05), whereas the *P* value of the Child–Pugh score, Hb, WBC, AST, Plt, Alb, Serum AFP > 400 ng/mL, tumor size, TACE+ in univariate analysis was less than 0.001. We also included HIV+ in the univariate analysis, and the results did not show a statistical impact on the prognosis of the disease, *P* = 0.814. We then conducted a multivariate analysis of these factors, and the results are shown in Table 2. The statistically significant factors were Child–Pugh score, WBC, AST, serum AFP > 400 ng/mL, tumor size, and TACE+. Of these, TACE+ was an independent protective factor. The relative risks (RR) of these factors were also calculated. The factors for RR > 1.2 or < 0.8 were Child–Pugh score, serum AFP > 400 ng/mL, and TACE+ ; the others had little correlation with the prognosis.

**Table 1:** The variables in the univariate analysis for the BCLC-B stage.

Variables	All included patients (n = 1464)		HIV(+) patients(n = 21)	
	Relative risk (95% CI)	P	Relative risk (95% CI)	P
Age (mean ± SD, years)	1.001 (0.996–1.007)	0.643	1.041 (0.987–1.098)	0.139
Male	1.051 (0.873–1.265)	0.598	0.485 (0.145–1.625)	0.241
BMI (mean ± SD, kg/m^2^)	0.974 (0.948–1.000)	0.054	1.007 (0.732–1.386)	0.964
HBsAg (+)	0.839 (0.695–1.012)	0.066	0.523 (0.156–1.757)	0.295
Child–Pugh score (mean ± SD)	1.417 (1.287–1.561)	< 0.001	0.609 (0.226–1.642)	0.327
TB (mean ± SD,μmol/L)	1.004 (1.001–1.007)	0.018	1.052 (0.971–1.140)	0.215
HB (mean ± SD,g/dl)	0.993 (0.989–0.996)	< 0.001	0.987 (0.968–1.007)	0.209
WBC (mean ± SD ,109/L)	1.121 (1.080–1.164)	< 0.001	1.018 (0.579–1.791)	0.950
CRE (mean ± SD, μmol/L)	0.998 (0.995–1.002)	0.331	0.979 (0.953–1.006)	0.125
ALT (mean ± SD, u/L)	1.001 (1.000–1.003)	0.015	0.971 (0.944–0.998)	0.037
AST mean ± SD, u/L)	1.003 (1.002–1.004)	< 0.001	0.973 (0.948–0.999)	0.039
Plt (mean ± SD ,109/L)	1.002 (1.001–1.003)	< 0.001	0.989 (0.978–1.001)	0.070
Alb (mean ± SD ,g/L)	0.959 (0.945–0.973)	< 0.001	1.094 (0.967–1.237)	0.154
PT (mean ± SD , s)	1.097 (1.055–1.141)	< 0.001	0.827 (0.422–1.620)	0.579
Serum AFP > 400 ng/mL	1.694 (1.468–1.955)	< 0.001	0.522 (0.110–2.470)	0.413
Number of tumor ≥ 2	0.979 (0.831–1.153)	0.801	1.404 (0.305–6.473)	0.663
Tumor size (mean ± SD , cm)	1.074 (1.055–1.093)	< 0.001	1.782 (0.774–4.104)	0.174
TACE (+)	0.422(0.347–0.514)	< 0.001	NA	NA
HIV(+)	0.934 (0.528–1.652)	0.814	NA	NA
CD3	NA	NA	1.000 (0.998–1.001)	0.882
CD4	NA	NA	1.001 (0.998–1.004)	0.621
CD8	NA	NA	0.999 (0.997–1002)	0.552

*BMI* body mass index, *HBsAg* Hepatitis B surface antigen, *TB* total bilirubin, *AFP* alpha-fetoprotein, *PT* prothrombin time, *WBC* white blood cell, *Cre* creatinine, *HB* hemoglobin, *Plt* platelet, *Alb* albumin.

**Table 2:** The variables in the multivariate analysis for the BCLC-B stage.

All included patients (n = 1464)			HIV(+) patients(n = 21)		
Variables	Relative risk (95% CI)	P	Variables	Relative risk (95% CI)	P
Child–Pugh score (mean ± SD)	1.250 (1.129–1.384)	< 0.001	Plt	0.983 (0.968–0.999)	0.038
WBC (mean ± SD ,109/L)	1.106 (1.065–1.149)	< 0.001	ALT	0.966 (0.934–0.999)	0.043
AST mean ± SD, u/L)	1.002 (1.001–1.002)	< 0.001	Tumor size (mean ± SD , cm)	4.009 (1.468–10.948)	0.007
Serum AFP > 400 ng/mL	1.499 (1.293–1.739)	< 0.001			
Tumor size (mean ± SD , cm)	1.050 (1.031–1.069)	< 0.001			
TACE (+)	0.480 (0.392–0.588)	< 0.001			

The variables of P < 0.1 in single factor analysis were included in multivariate analysis.

*Plt* platelet, *Alb* albumin; *AST* aspartate aminotransferase; *ALT* alanine aminotransferase, *AFP* alpha-fetoprotein, *WBC* white blood cell.

However, in order to independently study some specific indicators of HIV patients, we conducted a single-factor and multi-factor analysis of HIV patients. Of the 21 HIV patients included, CD4 of 8 patients was lower than 200/μL, and all patients received anti-virus treatment for HIV before operation. The mean values of CD3, CD4, and CD8 in the experimental group were 832.10 ± 422.61, 279 ± 174.70, and 493.38 ± 253.47, respectively. Preoperative CD3, CD4, and CD8 values were added to the univariate analysis of the HIV population. In the multivariate analysis of the HIV group, we found that Plt count, ALT, and tumor size had statistical significance (*P* = 0.038, 0.043, and 0.007, respectively). The RR of the first two items was greater than 0.96 and less than 1.0, but of the last, 4.009. This suggests that tumor size may be an independent risk factor in HIV+ patients.

### Baseline demographic and disease features

A total of 21 BCLC-B HCC patients were diagnosed with HIV infection. The average age at the time of HIV diagnosis was 48.07 ± 12.03 years, and the estimated exposure age was 44.57 ± 10.99 years. Groups were divided into three groups after matching as shown in Fig. S1. It is worth pointing out that because all HIV+ BCLC-B HCC patients in our hospital received surgery or TACE treatment, and no HIV+ BCLC-B HCC patients only received anticancer drugs, the second control group we chose was HIV− BCLC-B HCC patients only received medication. When they first diagnosed HCC in our hospital, the mean age of all 1464 patients included was 54.01 ± 13.03 years, of non-HIV patients was 54.07 ± 13.04 years, and of HIV+ patients was 50.24 ± 12.10 years (95%Cl, -9.45 to 1.79, *P* = 0.181). Of all patients included, 84.6% were positive for HBV. Of the 21 HIV+, 17 patients had HBV infection; 12 were vertical transmissions, and the average age at the time of HBV diagnosis was 42.60 ± 13.87 in the remaining 5 cases. Using the logarithmic function “lg” to deal with HBV viral load, our data showed that the mean lg viral load of 7 patients from 21 HIV+ patients who did not receive antiviral treatment of HBV was 4.43 ± 2.31, while that of the all non-HIV patient enrolled was 5.47 ± 5.40 (95%Cl, -5.06 to 2.98, *P* = 0.612). In the matched cohort, only one patient in the HIV+ TACE+ group had HCV, while no HCV-positive patients were found in the post-matched HIV− TACE+ and HIV− TACE− groups. We compared HIV+ TACE+ with HIV− TACE+ and HIV− TACE− according to the one-to-two nearest-neighbor match. The baseline features of HIV+ TACE+, HIV− TACE+ and HIV− TACE− cohort are presented in Table 3. After matching was performed, the baselines of each group consistent and the *P* value was > 0.1.

**Table 3:** Baseline characteristics.

Variables	HIV(+) TACE (−) (n = 21)	HIV (−) TACE (+) (n = 42)	P value	HIV(−)TACE(−) (n = 42)	P value
Age (mean ± SD, years)	50.24 ± 12.10	48.38 ± 12.25	0.570	50.25 ± 12.20	0.997
Male (%)	16(76.2%)	31(73.8%)	0.838	35(83.3%)	0.734
BMI ( mean ± SD, kg/m^2^)	22.30 ± 2.17	22.33 ± 2.18	0.103	22.06 ± 1.50	0.618
HBsAg ( ±)	15(71.4%)	33(78.6%)	0.530	32(76.2%)	0.682
Child–Pugh score (mean ± SD)	5.52 ± 0.60	5.64 ± 0.76	0.534	5.45 ± 0.71	0.693
TB (mean ± SD, μmol/L)	16.60 ± 7.95	16.75 ± 8.12	0.946	18.29 ± 7.69	0.420
HB (mean ± SD, g/dl)	126.81 ± 28.00	124.88 ± 21.79	0.765	129.24 ± 22.19	0.709
WBC (mean ± SD ,109/L)	6.42 ± 0.95	6.01 ± 2.40	0.335	6.54 ± 1.68	0.723
CRE (mean ± SD, μmol/L)	65.73 ± 25.54	63.62 ± 11.96	0.722	68.98 ± 15.30	0.531
ALT (mean ± SD, u/L)	44.52 ± 28.86	39.52 ± 27.24	0.503	45.88 ± 49.62	0.908
AST mean ± SD, u/L)	63.05 ± 49.00	62.71 ± 101.82	0.989	69.29 ± 73.83	0.728
Plt (mean ± SD, 109/L)	106.19 ± 51.80	109.76 ± 57.07	0.810	106.40 ± 51.20	0.988
PT (mean ± SD, s)	13.74 ± 1.11	13.32 ± 3.34	0.577	13.37 ± 1.44	0.310
Alb (mean ± SD, g/L)	37.96 ± 4.31	37.01 ± 6.28	0.537	38.56 ± 4.83	0.632
Serum AFP > 400 ng/mL (%)	4(19.0%)	8(19.0%)	0.734	13(31.0%)	0.316
Number of tumor ≥ 2 (%)	17(81.0%)	33(78.6%)	0.912	33(78.6%)	0.912
Tumor size (mean ± SD , cm)	4.43 ± 0.73	4.42 ± 2.2	0.980	4.83 ± 2.33	0.319

*BMI* body mass index, *HBsAg* hepatitis B surface antigen, *TB* total bilirubin, *AFP* Alpha-fetoprotein, *PT* prothrombin time, *WBC* white blood cell, *Cre* creatinine, *HB* hemoglobin, *Plt* platelet, *Alb* albumin.

### Survival of HIV+ patients and their matched controls

**Figure 1:**
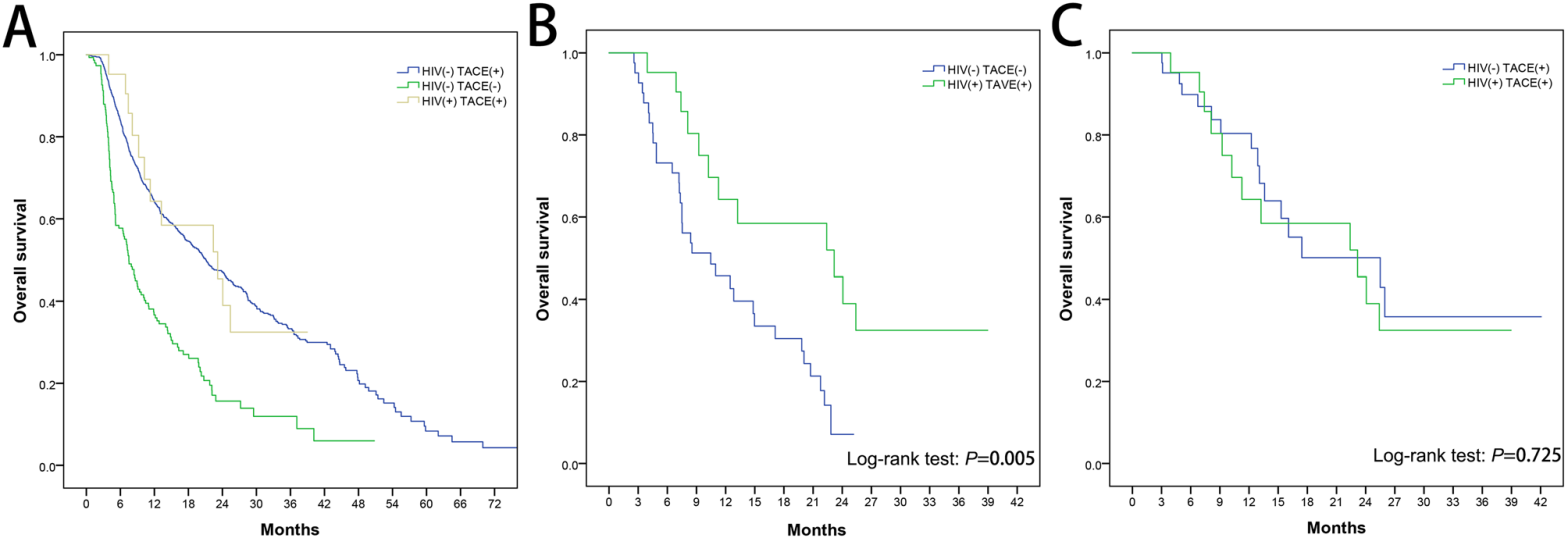
Kaplan Meier curve analysis of overall survival. (**A**) Overall survival rate of HIV (+) TACE (+), HIV (−) TACE (+) and HIV (−) TACE (−) groups before matching (**B**) post-matched overall survival rate of HIV (+) TACE (+) and HIV (−) TACE (−) (**C**) post-matched overall survival rate of HIV (+) TACE (+) and HIV (−) TACE (+).

Figure 1 shows the overall survival rate for the above each group containing before matching and after matching. After matching, the one-year survival rates of HIV− TACE+, HIV− TACE−, and TACE+ HIV+ were 76.5%, 45.7% and 64.3% (HIV+ TACE+ vs. HIV− TACE−, *P* = 0.097; HIV+ TACE+ vs. HIV− TACE+, *P* = 0.453), while 2-year survival rates were 50.0%, 7.1%, and 45.5%, respectively (HIV+ TACE+ vs. HIV− TACE−, *P* = 0.004; HIV+ TACE+ vs. HIV− TACE+, *P* = 0.790). The median survival time of HIV− TACE+, HIV− TACE−, and TACE+, HIV+ was 24.22 ± 2.95 months (95%Cl, 18.439 to 30.009), 12.29 ± 1.22 months (95%Cl, 9.909 to 14.675), and 22.54 ± 3.03 months (95%Cl, 16.598 to 28.491) respectively.

### Meta-analysis

**Figure 2:**
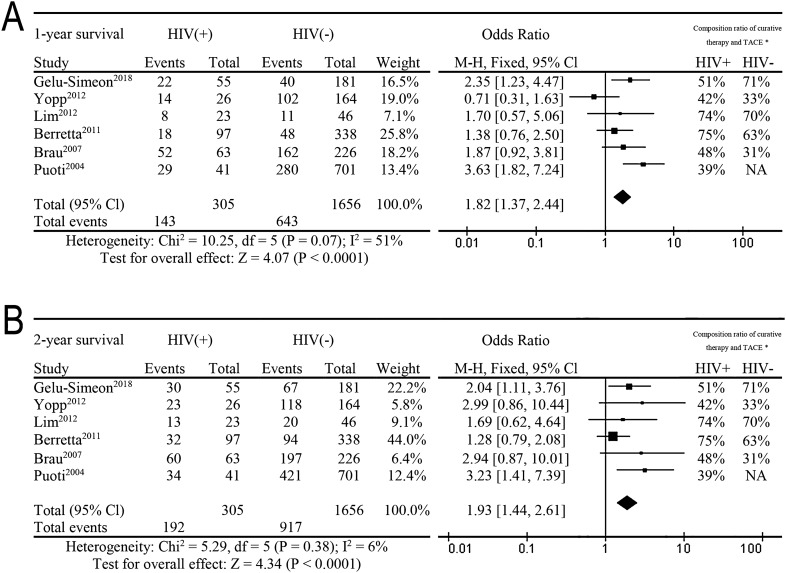
The overall meta-analysis comparing the overall survival between HCC patients with HIV and without HIV. * Composition ratio of curative therapy and TACE: (Curative therapy + TACE)/ the total number of HIV+ patients or HIV− patients. (**A**) Based on one year survival rate. (**B**) Based on two year survival rate.

Total searches yielded 353 entries. Ultimately 6 studies were selected, which were comparable retrospective studies. All included BCLC staging, treatment modalities and overall survival analysis, as shown in Fig. 2. The prognosis of HIV+ HCC patients was significantly lower than that of HIV− HCC patients regarding the 1- and 2-year survival rates.

In two of these studies, we found that the prognosis of HIV+ patients was significantly worse than that of HIV patients in Fig. 2. By calculating the proportion of curative therapy or TACE in the six studies, we found that in the study of Gelu-Simeon et al.^10^ HIV− patients had a significantly higher proportion of curative therapy or TACE (51% vs. 71%, *P* = 0.007) than other studies. In the study of Puoti et al., there is a lack of data on the proportion of HIV+ patients treated with radical therapy and TACE. Meta-analysis showed that the poor prognosis of HIV+ patients may be due to the low proportion of HIV+ patients receiving curative treatment or TACE in most studies.

## Discussion

### The lower proportion of HIV patients receiving surgical treatment in China

There are six related studies on the treatment of HIV+ HCC patients from the United States, France, Italy and other European countries. Overall, the rate of curative and TACE treatments was lower in HIV+ than HIV− patients (Fig. 2). Currently, the above reports on HIV+ HCC patients are mainly concentrated in Europe or America, where HIV treatment is relatively mature, while there are no reports on HIV+ HCC patients in Asia and Africa. We are the largest center for HIV patients in Southwest China. Through nearly 20 years of clinical treatment, nearly 2,000 patients with HIV have been treated annually. Approximately 3,000 of these have been treated surgically, of whom over 800 were treated in 2018. However, only 30 patients with HIV+ have been diagnosed with HCC, including 21 patients with stage BCLC-B, and 3 patients with stage BCLC-A. Pinato et al. published the largest HIV+ clinical study in 2018, involving 387 patients, of whom 33% were BCLC-A and 18% BCLB-B^11^. The reasons for the high rate of BCLC-B patients in our hospital were that most of them received no surgical treatment before they were transferred to our hospital; and that during this period, their staging also progressed from A to B. Although the current law of our country stipulates that HIV patients should enjoy an equal right to medical treatment, in fact HIV patients are mainly concentrated in specialized infectious disease hospitals. These are deficient in surgical technology compared with large general hospitals. Surgical resection and liver transplantation are the main treatment methods for hepatocellular carcinoma and have a good curative effect, but due to the particularity of HIV, the implementation of treatment methods is often inadequate.

### Occurrence and development of HCC in HIV+ patients

The shorter onset time of HCC in HIV patients is still the focus of research and discussion^12^. Because of the shared route of transmission and the immunosuppressive characteristics of HIV, HIV+ patients are more likely to develop HBV or HCV infection^13–15^. Our data show that the median age of HIV infected patients at first diagnosis of HCC in our hospital was 50.24 + 12.10 years, while that of the overall HIV− population was 54.07 ± 13.04 years (*P* = 0.181). The overall patients we included, including non-HBV HCC patients, were no younger than HIV HCC patients. This difference may be due to the first time of HIV exposure. Puoti et al.^12^ reported that the possible age of exposure is during patients’ twenties while our center's HIV+ patients, the first diagnosis of HIV antibody positivity is 48.07 ± 12.03 years, and possible exposure age was 44.57 ± 10.99 years.

In addition, the weakening of immune response caused by HIV will increase the viral load and accelerate the progression of hepatitis cirrhosis and hepatocellular carcinoma through the carcinogenic characteristics of HBV or HCV themselves. Since anti-hepatitis B drugs have a significant impact on viral load, we chose HIV+ HBV co-infected HCC patients who did not receive HBV antiviral therapy at the time of consultation, to compare with HIV− HBV HCC patients. Our results showed that there was no significant difference in viral load and onset time between HIV HBV co-infected HCC patients and HIV− HBV infected HCC patients. (*P* = 0.612).

However, due to HIV-induced immunosuppression, the reduced immune response to HBV or HCV may actually reduce liver inflammation and injury, thereby slowing the progression of cirrhosis and cancer. Meta-analysis^16^ showed that Fibrosis-4 index (FIB-4) was a more accurate non-invasive method for evaluating the degree of fibrosis. Because biopsy results cannot be obtained at the TACE procedure itself, we chose FIB-4 to reflect the degree of liver fibrosis. Our data showed that the degree of hepatic fibrosis in 21 HIV+ patients was 6.06 ± 5.00 and in 1443 non-HIV patients was 5.14 ± 6.03 (*P* = 0.486, 95% CI -1.67 to 3.51).

Viral load, hepatic fibrosis index and onset time did not show a significant impact on HIV positive patients. However, the degree of liver fibrosis has not been determined at biopsy, too few cases of viral load were measured and the short interval between HIV exposure time and HCC detection time may have some impact on these results. We suggest that a prospective multi-center study be undertaken specifically to validate these indicators.

### Prognostic risk factors in patients with HCC

It is still controversial whether HIV combined with hepatitis virus may increase the risk of HCC occurrence^13,17,18^; however, what can be confirmed is that HAART based on survival analysis is currently recognized as an independent protective factor for HIV co-infected patients^19–22^. Since the introduction of HAART therapy, there have been several reports from HIV+ and HIV− clinical control studies^10,12,23–26^. The current consensus is: 1. BCLC grade is the main risk factor affecting prognosis of HIV+ HCC patients; 2. The prognosis of untreated patients is significantly worse than that of patients receiving treatment. Our univariate and multivariate analyses showed that HIV+ was not a prognostic risk factor for BCLC-B patients. For all patients of BCLC-B stage, our data show that TACE treatment has the most significant protective significance. In addition, combined RR values, AFP and Child–Pugh Score are the most important risk factors. This may be due to that BCLC-B staging limited liver function, size and number of tumors.

The largest single-arm study of 387 HIV+ HCC patients in a multi-center trial showed that CD4 is a potential prognostic factor for the survival of HIV+ HCC patients^11^. In order to study some specific indicators of HIV infection, we conducted single and multi-factor analyses of HIV patients to assess the significance of varying levels of CD3, CD4 and CD8. Our central data show that the levels of CD3, CD4, and CD8 were not considered to be a prognostic factor. However, three of the eight patients whose CD4 was below 200/μL had opportunistic bacterial infections, and the CD4 level also remained low, after surgery. None of the 13 patients whose CD4 was greater than 200/μL had opportunistic bacterial infections (*P* = 0.042). The HIV RNA is also considered as a potential independent predictor. The three patients with opportunistic bacterial infection had HIV replication > 5 × 10^2^, 2.11 × 10^3^, 1.91 × 10^6^, while 5 patients in the other 18 patients without opportunistic bacterial infection had HIV RNA levels > 5 × 10^2^ before operation (*P* = 0.274). From the statistical results of *P*-value, the prediction value of CD4 for postoperative opportunistic infection was better than that of HIV RNA. Unfortunately, due to the limited number of cases, we were unable to conduct a control study to guide whether CD4 count or HIV RNA is more predictive for the postoperative opportunities. Interestingly, we found that Plt count and AST are protective factors for HIV patients, but RR is of less significance; this may reflect that there were only 21 samples, and we look forward to a larger control study to verify this.

Overall, the AFP, Child–Pugh score, and tumor size may increase with the progression of the disease in both the general population and the independent HIV population. Therefore, TACE should be performed as early as possible for BCLC-B HCC patients, including HIV-positive patients, to improve prognosis.

### Survival time of HIV patients with HCC

The prognosis of HIV+ and HIV− patients is still inconsistent. The 5-year survival rate is generally believed to be worse for HIV+ than for HIV−^25^. Gelu-Simeon et al.^10^ is currently the largest multicenter study, showing that the 1-and 2-year survival rates of HIV+ patients are significantly worse than that of HIV− patients. Pinato et al. mainly studied the survival of HIV positive patients with different albumin levels. There was no HIV negative patients as control group, so we did not include their research in meta-analysis^11^. From Fig. 2, we can see that only 2 of the 6 studies have significant poor prognosis in patients with HIV+. In addition, we found that the proportion of HIV− patients receiving TACE treatment in one of the above two studies is significantly higher than that in HIV+ patients (71% vs. 51%, *P* = 0.007), while another one is lack of relevant data. Considering the low proportion of HIV+ patients receiving curative treatment, Lim et al.^24^ proposed a 1:2 matching method to control the deviation caused by the proportion of treatment modalities. This report had the smallest difference in the composition ratio between HIV+ group and HIV− treatment mode (73.9% vs.69.6%, *P* = 0.704). As a result, there was no statistical difference in the overall survival rate. In the study of Berretta et al.^25^ the curative or TACE treatment of HIV+ patients accounted for 75% and of the HIV− group accounted for 63%; the overall survival rate of the HIV+ group was poor, but there was no significant difference in survival rates at 1 and 2 years. In other studies where the ratio of curative and TACE treatment was slightly different between HIV+ and HIV− groups, there was no statistical difference in 1 or 2-year prognosis.

Overall, through meta-analysis of previous studies, the survival rate of HIV+ patients is still lower than that of HIV− patients. Previous studies show that BCLC-A patients account for a higher proportion of HIV+ than HIV− patients, while HIV positive patients receive a lower proportion of the treatment recommended by BCLC. As a result, this may also be the cause of the poor prognosis of HIV+ patients. Based on the shortcomings of previous studies, we hope to be able to strictly match HIV+ and HIV—patients with the data of our center over 16 years. Matching will strictly control the selection of patients in a single stage, i.e. BCLC-B stage, and treatment guidelines, i.e. recommended treatment TACE, as well as preoperative conditions. Since the median survival rate of BCLC-B patients is about 2.5 years, it would be more important to ensure the 1 and 2-year survival rate of these patients. Our data show that there is no significant difference in 1-year and 2-year survival rates between HIV+ and HIV− patients, and they are much higher than those without surgical treatment. In conclusion, we believe that TACE treatment should be actively carried out for HIV+ patients in the BCLC-B stage.

### Prospects and limitations

HIV therapy is still developing in China. In order to create a mature medical system for HIV-HCC patients and to realize equal surgical opportunities for HIV and ordinary patients, policies such as HIV surgical treatment still need to be improved at the national level, followed by appropriate education for doctors and patients. The retrospective design should be acknowledged as a limitation to our work. Because of the retrospective study, it is difficult to avoid the selective bias, even when using a case–control study design based on propensity score matching. Furthermore, the case–control matching may lead to other selective biases due to unmeasured patient characteristics which may have influenced outcomes. There were only 21 HIV+ patients undergoing TACE in the BCLC-B stage; however, this study is—to our knowledge—the largest case control study of survival outcomes of BCLC-B stage HIV patients who underwent TACE.

## Conclusion

In many countries where surgical treatment for HIV HCC patients is not yet mature, drug therapy currently is the ultimate treatment for many. Because TACE has lower exposure risk and implementation conditions than large-scale surgery, and because the survival rate of HIV+ is similar to that of HIV− patients, we suggest that TACE treatment should be actively carried out for BCLC-B HIV HCC patients as early as possible.

## Supplementary Information

Supplementary Information.
