# Supplementary material for: Outcome of TACE treatment in HIV infected patients with hepatocellular carcinoma
Source: Sci Rep. 2021 Jan 12;11:696. doi: 10.1038/s41598-020-80311-3 (PMC7804028; doi:10.1038/s41598-020-80311-3)
Supplement: Supplementary file 1 — Supplementary Information. [file 41598_2020_80311_MOESM1_ESM.pdf]

Outcome of TACE Treatment in HIV Infected Patients with Hepatocellular Carcinoma

LingXiang Kong[1*], Guo Wei[2*], Tao Lv[1], Li Jiang[1], Jian Yang[1], Yong Zhao[2], Jiayin Yang[1]

[1]Department of Liver Surgery, West China Hospital of Sichuan University, Chengdu, Sichuan Province, China.

[2] Department of General Surgery, Chengdu Public Health Clinical Medical Center, Chengdu, Sichuan Province, China.

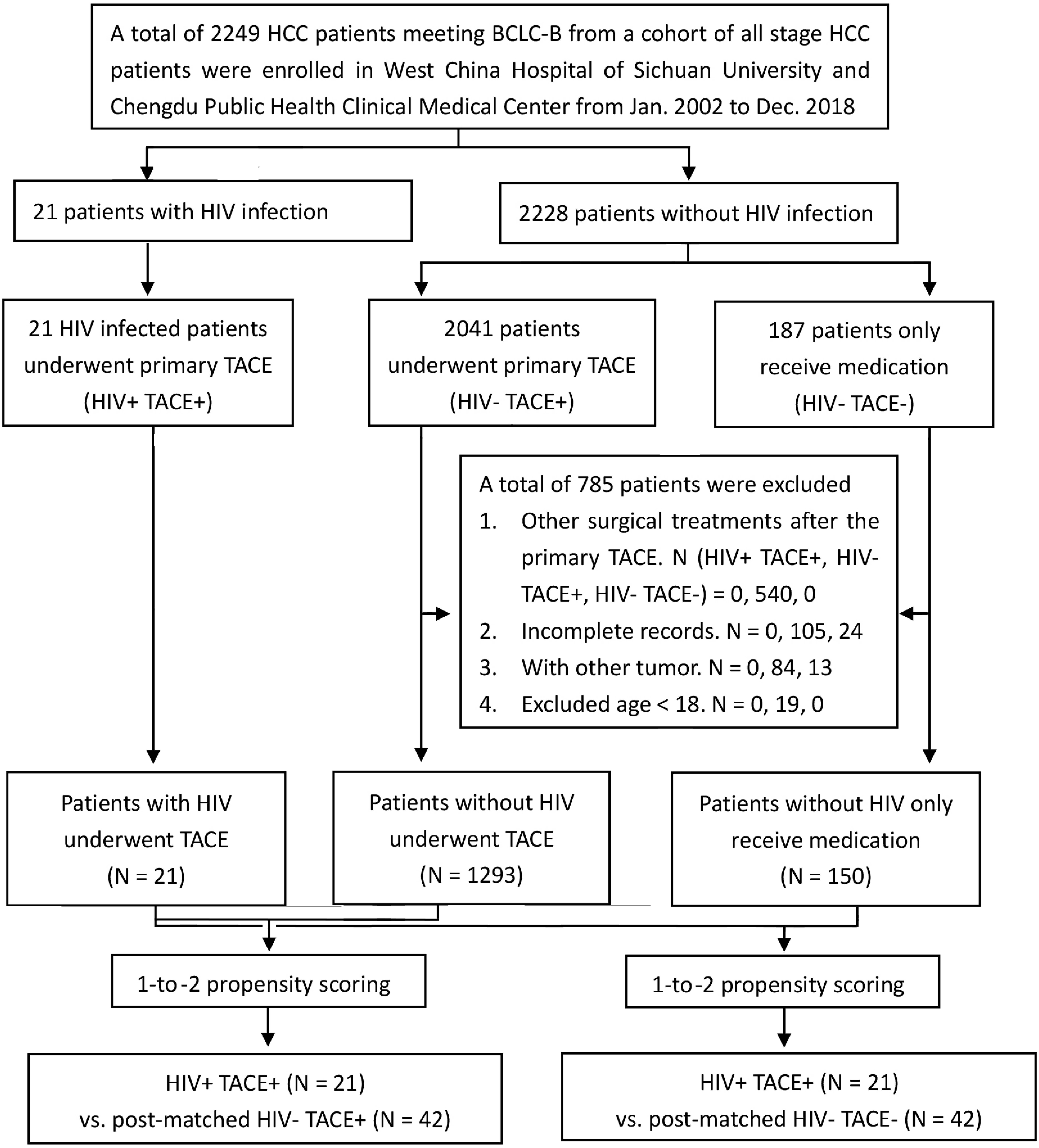

Fig.S1 Flow of study participants.
